# The North American mitochondrial disease registry

**DOI:** 10.20517/jtgg.2020.12

**Published:** 2020-04-28

**Authors:** Xiomara Q. Rosales, John L. P. Thompson, Richard Haas, Johan L. K. Van Hove, Amel Karaa, Danuta Krotoski, Kristin Engelstad, Richard Buchsbaum, Salvatore DiMauro, Michio Hirano, The North American Mitochondrial Disease Consortium

**Affiliations:** 1 Department of Neurology, Columbia University Medical Center, New York, NY 10032, USA.; 2 Department of Biostatistics, Mailman School of Public Health, Columbia University, New York, NY 10032, USA.; 3 Departments of Neurosciences and Pediatrics, University of California at San Diego, San Diego, CA 92093, USA.; 4 Department of Pediatrics, University of Colorado School of Medicine, Aurora, CO 80045, USA.; 5 Genetics Unit, Massachusetts General Hospital, Boston, MA 02114, USA.; 6 Eunice Kennedy Shriver National Institute of Child Health and Human Development, National Institutes of Health, Bethesda, MD 20817, USA.

**Keywords:** Mitochondrial disease, registry, consortium, rare disease

## Abstract

**Aim:**

The North American Mitochondrial Disease Consortium (NAMDC) comprises a network of 17 clinical centers with a mission to conduct translational research on mitochondrial diseases. NAMDC is a part of the Rare Disease Clinical Research Network (RDCRN) and is funded by the National Institutes of Health. To foster its mission, NAMDC has implemented a comprehensive Mitochondrial Disease Clinical Registry (hereafter NAMDC Registry), collected biosamples deposited into the NAMDC Biorepository, defined phenotypes and genotypes of specific disorders, collected natural history data, identified outcome measures, characterized safety and long-term toxicity and efficacy of promising therapies, and trained young investigators interested in patient-oriented research in mitochondrial disease.

**Methods:**

Research conducted by NAMDC is built on the foundation of the Clinical Registry. Data within the registry are encrypted and maintained in a centralized database at Columbia University Medical Center. In addition to clinical data, NAMDC has established a mitochondrial disease biorepository, collecting DNA, plasma, cell, and tissue samples. Specimens are assigned coded identifiers in compliance with all relevant regulatory entities and with emerging NIH guidelines for biorepositories. NAMDC funds two pilot projects each year. Pilot grants are small grants typically supporting an early stage concept to obtain preliminary data. Pilot grants must enhance and address major issues in mitochondrial medicine and specific areas of need for the field and for the successful outcome of NAMDC. The grant selection process is facilitated by input from multiple stakeholders including patient organizations and the strategic leadership of NAMDC. To train new mitochondrial disease investigators, NAMDC has established a Fellowship Program which offers a unique training opportunity to senior postdoctoral clinical fellows. The fellowship includes a 6-month period of intensive training in clinical trial methodology through the Clinical Research Enhancement through Supplemental Training program and equivalent programs at the other sites, along with rotations up to 3 months each to two additional consortium sites where a rich and varied training experience is provided. Nine core educational sites participate in this training program, each offering a summer grant program in mitochondrial medicine funded by our NAMDC partner the United Mitochondrial Disease Foundation (www.umdf.org). All clinical research in NAMDC depends on the participation of mitochondrial disease patients. Since individual mitochondrial disorders are often extremely rare, major communication and recruitment efforts are required. Therefore, NAMDC has forged a very close partnership with the premier patient advocacy group for mitochondrial diseases in North America, the United Mitochondrial Disease Foundation (UMDF).

**Results:**

The NAMDC Registry has confirmed the clinical and genetical heterogeneity of mitochondrial diseases due to primary mutations in mitochondrial DNA or nuclear DNA. During the 8 years of this NIH-U54 grant, this consortium, acting in close collaboration with a patient advocacy group, the UMDF, has effectively addressed these complex diseases. NAMDC has expanded a powerful patient registry with more than 1600 patients enrolled to date, a website for education and recruitment of patients (www.namdc.org), a NAMDC biorepository housed at the Mayo Clinic in Rochester, MN, and essential diagnostic guidelines for consensus research. In addition, eight clinical studies have been initiated and the NAMDC fellowship program has been actively training the next generation of mitochondrial disease clinical investigators, of which six have completed the program and remain actively involved in mitochondrial disease research.

**Conclusion:**

The NAMDC Patient Registry and Biorepository is actively facilitating mitochondrial disease research, and accelerating progress in the understanding and treatment of mitochondrial diseases.

## INTRODUCTION

Mitochondrial diseases due to OxPhos defects and caused by mutations in either the mitochondrial or the nuclear genome are an exceptionally heterogeneous group of disorders. Some are confined to the central or peripheral nervous system (encephalopathy, peripheral neuropathy, and myopathy) but most are multisystemic. Although clinical severity varies, often within families or within cohorts of patients harboring the same pathogenic mutation, by and large these are progressive and often crippling disorders, causing weakness, exercise intolerance, fatigue, ataxia, seizures, mental retardation, dementia, hearing loss, blindness, parkinsonism, strokes, peripheral neuropathies, and premature death. Because of the clinical heterogeneity and the frequent multisystemic involvement, mitochondrial diseases present formidable diagnostic challenges. When accurately diagnosed, they pose even more formidable therapeutic difficulties, as there are very few disease-modifying therapies, and symptomatic therapies, often only anecdotally reported, and are only partially effective.

There is a vibrant international research community engaged in the study of the mechanisms and clinical characterization of mitochondrial diseases and the search for better treatments. However, since there is a large number of distinct mitochondrial diseases, each with relatively low incidence, it has been difficult to assemble sufficiently large datasets or patient groups for research studies and clinical trials.

**Figure 1.**
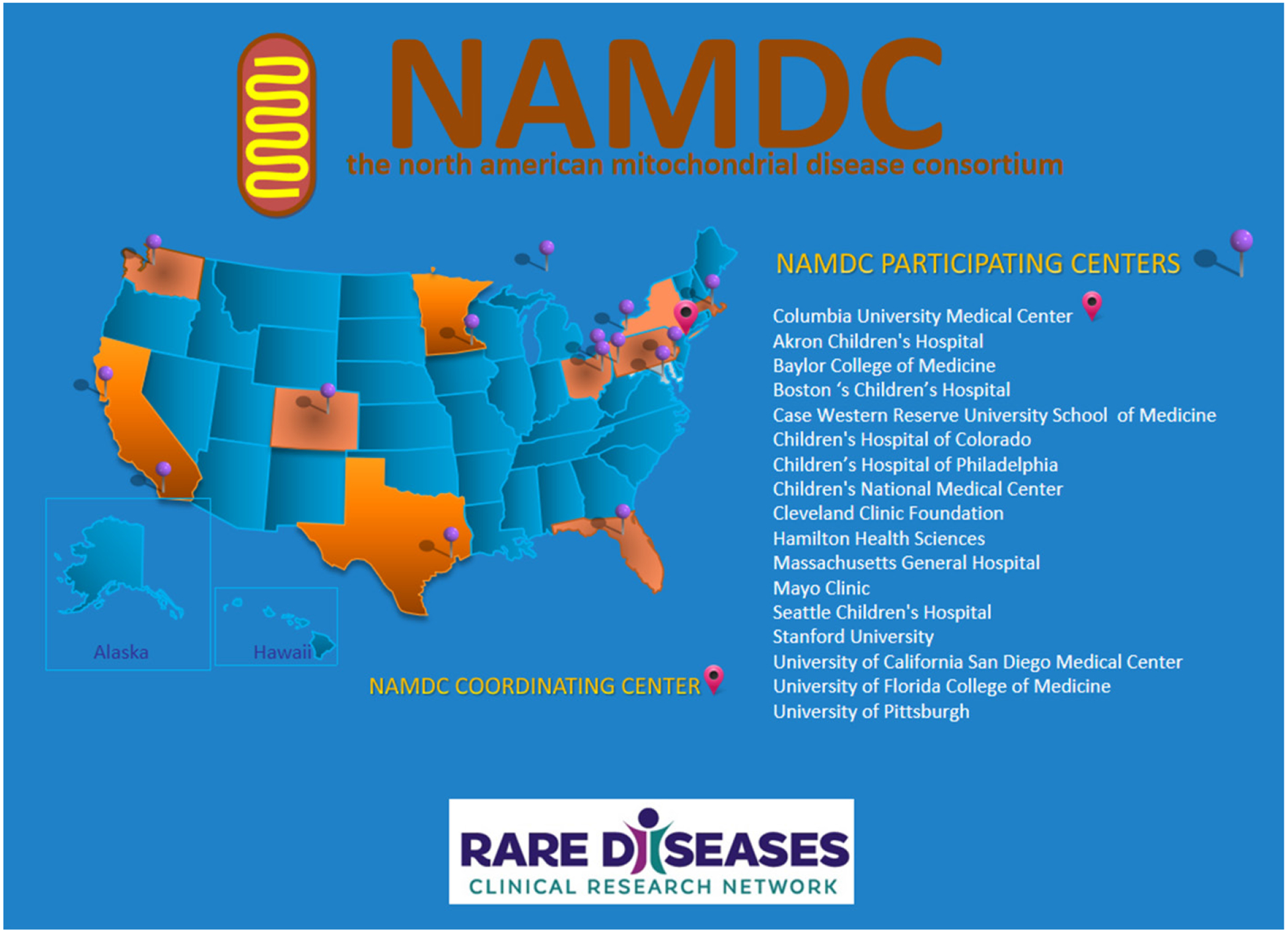
NAMDC Participating Centers. NAMDC: North American Mitochondrial Disease Consortium

The North American Mitochondrial Disease Consortium (NAMDC) was established to create a network of clinicians and clinical investigators in North America who follow sizeable numbers of patients with mitochondrial diseases and are interested in mitochondrial research. These “centers of excellence” collaborate with each other and with participating patients to collect vital information and conduct research on mitochondrial diseases. At present, NAMDC is collaborating with some of the leading researchers within the mitochondrial community at 17 academic institutions in United States and Canada [Figure 1].

By allowing researchers to aggregate patient data and biological samples from across all member sites, NAMDC eases some of the difficulties associated with studying these rare and diverse diseases. NAMDC is facilitating a number of clinical trials and natural history studies that would otherwise not be feasible due to insufficient sample size.

To join NAMDC, clinical investigators with expertise in mitochondrial diseases are required to submit an official request to the consortium’s Principal Investigator and Program Manager explaining how the new site will enhance the ability of the consortium to meet its goals and the commitment to comply will all data standards and study accountability as outlined in the RDRN Policies, Procedures, and Standards. The NAMDC Executive Committee then reviews the request and, if it is approved, forwards it to the Rare Disease Clinical Research Network (RDCRN) Board for final approval. At a minimum, NAMDC sites are required to fully participate in our Registry and Biorepository recruitment; to participate in other consortium activities, including monthly NAMDC and NAMDC Fellowship meetings; and to agree to abide by our research requirements and compliance goals.

## METHODS

### NAMDC registry

To collect information about the wide spectrum of mitochondrial diseases, NAMDC has designed and implemented a comprehensive NAMDC Mitochondrial Disease Clinical Registry that gathers baseline clinical, biochemical, and molecular genetic data and tracks the natural histories of the patients through the NAMDC Clinical Longitudinal study. All data in the NAMDC Registry are handled in a strictly confidential manner. All data are stored in encrypted databases, and all communications of data are password-protected and encrypted. Patient identifiers are stored separately from clinical data and are made available only to authorized users.

Patient enrollment may occur in person at one of the NAMDC member sites or through the Remote Recruitment program streamlined through the NAMDC Clinical Coordinating Center at Columbia University. The web-based remote enrollment (www.namdc.org) provides a patient-friendly platform that includes passcode access, online screening, electronic consent, and data capture for enrollment of eligible patients who reside far from any of the NAMDC participating sites and/or have difficulties traveling to the sites due to their disabilities.

The NAMDC Clinical Registry is also the base for developing the NAMDC Clinical Registry/Longitudinal Study to collect natural history data across the spectrum of mitochondrial diseases.

### NAMDC research diagnostic criteria

Because of the vast clinical and genetic heterogeneity of mitochondrial diseases, diagnosing these disorders is challenging, but critical to assess the natural histories, define prognoses, study pathogenic mechanisms, and develop clinical trials. To facilitate accuracy and uniformity of the diagnosis of mitochondrial disease, NAMDC investigators in various disciplines including Neurology, Internal Medicine, Pediatrics, Pathology, Clinical Genetics, Clinical Laboratory Medicine, and Biostatistics, have generated NAMDC Research Diagnostic Criteria for mitochondrial diseases with strict benchmarks for definite, suspected, or unlikely levels of diagnoses. Data derived from the registry have been the source for the development and refinement of the diagnostic criteria, which are being applied on a research basis to classify patients in the NAMDC Registry. The NAMDC research diagnostic criteria are outlined in a separate report that is currently in preparation for publication.

### NAMDC biorepository

At the time of consent into the NAMDC Patient Registry, patients are provided with the opportunity to submit samples to the NAMDC Biorepository. Samples of blood, urine, and other tissues are then collected and deposited into the NAMDC Biorepository. The NAMDC Biorepository is located at the Mayo Clinic Biospecimens Accessioning and Processing (BAP) Core in Rochester, Minnesota. Participation in the Biorepository is optional.

The NAMDC Clinical Registry, Research Diagnostic Criteria, and Biorepository are three pillars that form a strong foundation for multi-center collaborative mitochondrial disease research. The NAMDC registry includes a data-mining tool to facilitate access to de-identified data among NAMDC investigators as well as a consortium-style Master Service Agreement that allows transfer of de-identified biosamples among NAMDC sites, and to other external entities upon the appropriate approval.

### NAMDC pilot program

The Pilot Project Program is an important component of NAMDC, and supports 1–2 pilot projects annually. NAMDC pilot projects focus on biomarkers, diagnosis, natural history, and treatment of mitochondrial diseases. Thus, projects must be human subject studies, i.e., include patients, human cells, tissues, or body fluid samples. Pilot grants are often a first step for junior investigators, for whom the grant application is frequently the first formal funding application. The Pilot Core provides multiple mentoring steps for junior investigators including feedback on initial proposals (including unfunded proposals to improve the quality of the application), throughout the grant development process, and during project implementation. Mentoring is done by the NAMDC PI, Statistical PI, Pilot Core PI, local site PI, NAMDC Clinical Liaison, and additional collaborators as needed.

The NAMDC Pilot Program Committee is responsible for reviewing the pilot/new project proposals. Applicants for Pilot Projects must be NAMDC members. The NAMDC Pilot Project Committee recommends funding decisions that best meet the needs and priorities of the consortium and encourage its growth. Selection for funding is based upon information from written peer review and from the NAMDC PIs following oral presentation at the annual Face-to-Face meeting. Following the recommendation by the Pilot Program Committee, the NAMDC Executive Committee makes the ultimate decision as to what pilot/new projects are funded with input from the RDCRN and approval from the NIH officers. Multicenter studies are prioritized over single-site study projects. Applicants apply for funding for projects that occur during the 12-month window of the NAMDC yearly cycle of NIH funding. This means that applicants apply for projects that will be carried out from 1 September to 31 August of the following year. Grantees are expected to submit a brief quarterly report, a preliminary report presentation at the annual Face-to-Face meeting, and a final report within two months of the conclusion of the grant.

### The NAMDC career enhancement program

There is a severe shortage of physicians trained to diagnose and treat pediatric and adult patients with mitochondrial disease. The NAMDC Career Enhancement Program is designed to provide training in rare disease with a focus on mitochondrial medicine. The program consists of the following components:

The NAMDC Fellowship Program, which offers a unique training opportunity to senior postdoctoral clinical fellows to move on to the attending/assistant professor level in an academic setting as well-trained clinician scientists. The focus is on translational medicine, teaching of diagnostic expertise, and the development of clinical trials expertise. NAMDC aims to train the first of a new generation of clinician scientists who will be well equipped to move promising new treatments for mitochondrial disease into the clinical arena. Nine training sites are collaborating in this program. These sites - Columbia, San Diego, Seattle, Cleveland, Hamilton, CHOP, Baylor, University of Colorado, and Mayo Clinic - are all leading institutions in the field of mitochondrial medicine. World-renowned faculty are to be found at each site. Fellows participate in RDCRN training courses where they present a summary of their work.

The fellowship program is unified by telemedicine conferences led by the NAMDC Career Enhancement PI and involves all consortium sites with monthly video webinar conferencing. These conferences are well attended by faculty, students, and NIH representatives. RDCRN trainees are also invited to participate.

A Career Enhancement program is being developed to include: (1) a didactic core lecture series on Clinical Trial readiness to be available as an online lecture series; (2) the directors of the 10 Career Enhancement Sites to post a comprehensive series of lectures on management of mitochondrial disease; and (3) the Mitochondrial Medicine Society to develop a series of organ-specific mitochondrial disease lectures to be CME-certified by the United Mitochondrial Disease Foundation (UMDF) and made available online.

Funds provided by the UMDF will be used to attract young physicians, medical students, MD/PhD students, and recent graduates heading to medical school and the field of mitochondrial medicine. A committee of Site Directors and UMDF grant reviewers will review proposals. In addition, an annual retreat will take place on the day following the UMDF annual meeting where the awardees and the NAMDC fellow can interact with and present their work to the Site Directors.

### NAMDC administrative core

The Administrative Core of NAMDC provides critical organizational and strategic support to assure the overall success of the Program in accordance with NIH RDCRN program objectives. As the central hub and coordinating center for all NAMDC projects, the Administrative Core manages the interactions between multiple academic departments and between the Consortium and external organizations and institutions. The Core has an Overall Program Director/Principal Investigator, a Statistical Principal Investigator, a Clinical Team Liaison, a Bioinformatician, and an Administrative Coordinator, with ultimate responsibility for the Program’s scientific, clinical research, and training/educational operations. The NAMDC Executive Committee (EC) consists of these individuals plus the Chairs of the seven NAMDC Standing Committees (Data Use Committee, Biorepository Committee, Career Enhancement Program Committee, Pilot/New Project Program Committee, Publications Committee, Website Committee, and Diagnostic Committee), two NIH Representatives, and one Patient Advocacy Group representative. The EC meets regularly to assess the progress of NAMDC programs (protocols, initiatives, *etc.*), set the long-term strategic goals for the Consortium, and define the terms and rules by which the Consortium interacts with outside entities (Patient Advocacy Groups, non-NAMDC researchers, industry, *etc.*).

The Administrative Core also maintains three independent websites: (1) an internal website to manage internal documents and communications with NAMDC sites; (2) a patient remote enrollment website (www.namdc.org) recently expanded for remote enrollment in Latin America (www.namdc.org/sp); and (3) an external website coordinated with RDCRN to publicly communicate NAMDC’s mission and the availability of training opportunities and core services (https://www.rarediseasesnetwork.org/cms/namdc/).

## RESULTS

### NAMDC clinical registry and biorepository

**Figure 2.**
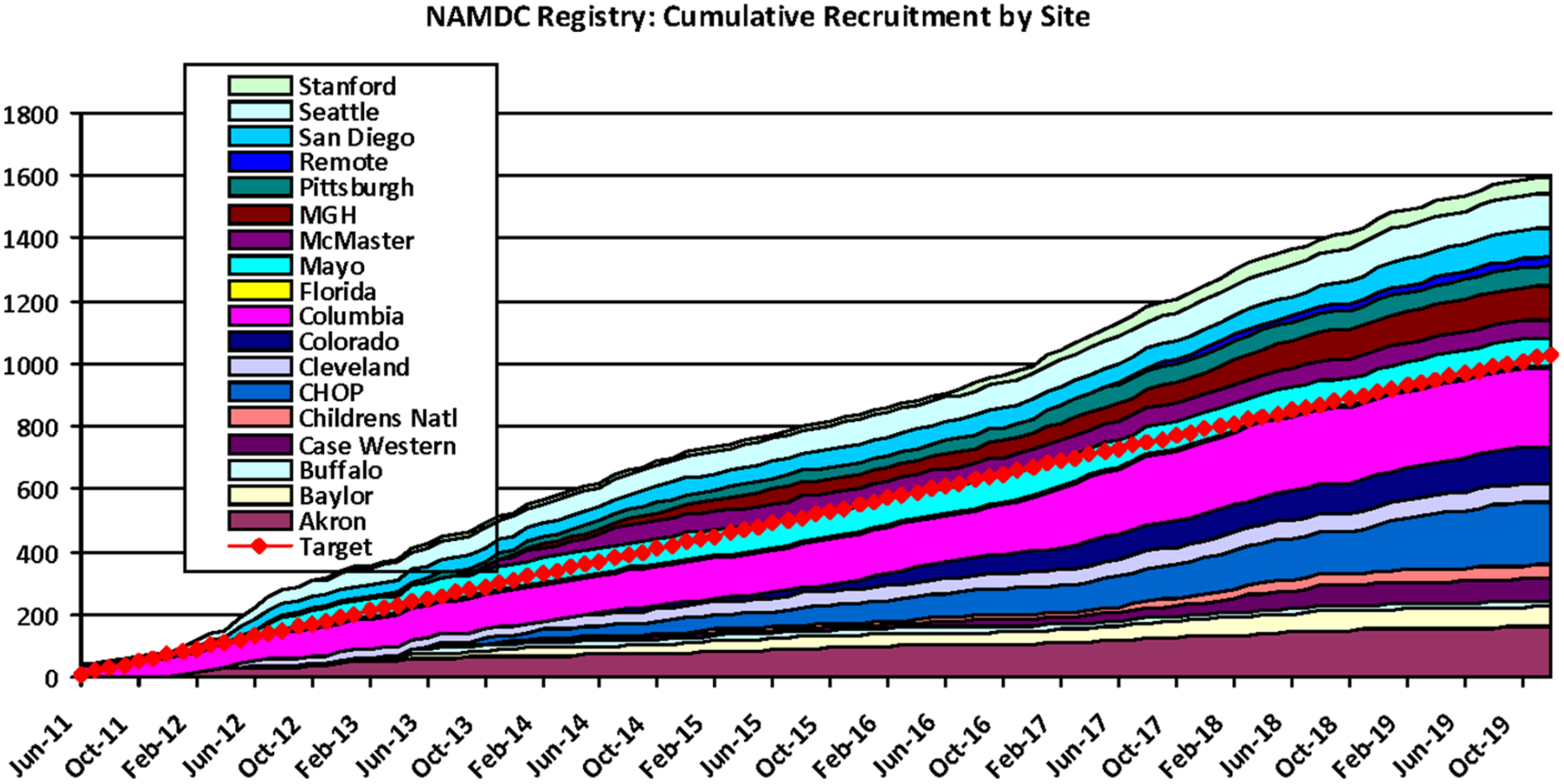
Actual vs. target recruitment. Data current as of December 31, 2019. Target Enrollment to Date: 1000; Enrolled to Date: 1634. NAMDC: North American Mitochondrial Disease Consortium

NAMDC is part of the Rare Diseases Clinical Research Network (RDCRN) funded by the National Institute of Health (NIH U54NS078059). NAMDC has established a network of 17 clinical centers of excellence to improve the diagnosis, natural history, and treatment of mitochondrial diseases [Figure 1]. NAMDC sites’ enrollment of mitochondrial disease patients is progressing steadily with more than 1600 patients enrolled to date [Figure 2]. The proportion of female participants in the registry population is 14.6% higher than male population and there is a notable under-representation of certain patient groups - individuals over age 65 year comprise only 3% and African American subjects represent only 2.4% (see Supplementary Materials). To enhance recruitment, the NAMDC Central Coordinating Center has also implemented a remote patient recruitment system which has been successfully enrolling domestic and international patients since 2018. Data from the NAMDC Clinical Registry [Table 1] have been analyzed, and and published in the journal of Neurology Genetics (Barca *et al*.^[1]^ 2020). A data-mining tool has been programmed to allow NAMDC site investigators to perform data queries for internal use. NAMDC is expanding the Registry to collect natural history data on all enrolled patients with select diseases. Additionally, the NAMDC Biorepository currently holds more than 330 biological samples as well as a virtual fibroblast of 185 cell lines. A Central IRB at Columbia University Medical Center has been established for this program.

### NAMDC pilot program

To date, nine pilot projects have been awarded. Some of them have been completed and published^[1–4]^, and others are in progress. The awarded projects focus on development of biomarkers, improving diagnostic tools, characterizing natural history, and testing therapies for mitochondrial diseases [Table 2].

### The NAMDC career enhancement program

The NAMDC Fellowship program has been highly successful with monthly webinar meetings and didactic training of six fellows. The first three trainees who completed the program have obtained faculty positions and are active mitochondrial disease clinician investigators. The three latest trainees are continuing their clinical training within the field of neurogenetics.

### NAMDC survey studies

In addition to the pilot studies, five patient surveys have been assessed through the NAMDC Registry: (1) attitudes of women carrying mitochondrial DNA mutations and healthy egg donors regarding oocyte nuclear transfer to prevent transmission of mtDNA disease; (2) nutritional supplement use in mitochondrial disease; (3) cardiovascular events in patients with metabolic diseases on chronic carnitine supplementation; (4) motivations and barriers for participation in clinical trials by individuals with mitochondrial diseases; and (5) the diagnostic odyssey of mitochondrial disease patients. Results of four of the five completed surveys have been published^[5–8]^. In addition, a survey of NAMDC investigators regarding the NAMDC Research Diagnostic Criteria has been completed and the results have contributed to the refinement of the NAMDC Diagnostic Criteria.

## DISCUSSION

Mitochondrial diseases are challenging because they may be the most diverse human disorders at every level: clinical, biochemical, and genetic. Some are confined to the nervous system but most are multi-systemic, often affecting the brain, heart, liver, skeletal muscle, kidney, endocrine, and respiratory systems. Although severity varies, largely these are progressive and often crippling disorders. They frequently cause weakness, exercise intolerance, fatigue, seizures, mental retardation, dementia, hearing loss, blindness, and premature death.

The challenge lies in the extraordinary clinical spectrum of mitochondrial diseases, which all too often leads practitioners to either underdiagnose (“what is this complex disorder?”) or to overdiagnose (“this disorder is so complex that it must be mitochondrial!”). The diagnostic difficulty is reflected in the diagnostic odyssey encountered by patients; in our survey study utilizing the RDCRN Contact Registry, subjects reported seeing, on average, more than eight clinicians before being given the diagnosis of mitochondrial disease^[4]^. Given this heterogeneity of mitochondrial disorders, it is essential to apply common, agreed-upon standards to the diagnosis and classification of patients. Further, given the variety of clinical and biochemical phenotypes, effective research on any given subset depends on aggregating relatively large numbers of patients.

These exigencies make clear that two prerequisites are necessary before effective clinical research can take place: (1) a clinical patient registry/longitudinal study to evaluate and expedite subsequent research with available patients; and (2) uniform diagnostic criteria for mitochondrial diseases. NAMDC was established with the goal of addressing these essential elements. After 8 years of continuous support of a U54 RDCRN cooperative agreement, NAMDC has built a basic infrastructure, established a robust clinical registry, expanded the NAMDC Biorepository, plans to implement a registry-wide Natural History study for more common mitochondrial disorders, continues support of pilot projects, and plans to expand the career enhancement program to train new mitochondrial disease investigators. Furthermore, NAMDC plans to collaborate in the international data-harmonization efforts aiming to provide users with access to a comparable view of data from global mitochondrial registries. As NIH funding of projects has a finite lifespan, NAMDC leadership is implementing innovative and cost-effective alternatives such as remote enrollment and is assembling a patchwork of funding sources by ensuring industrial support and strategic collaboration with the UMDF non-for-profit enterprise. Through this approach, NAMDC aims to build a critical infrastructure and performance level to support a resource-efficient and sustainable plan for future mitochondrial disease research in North America and in collaboration with international partners.

## Supplementary Material

supplementary materials

## Figures and Tables

**Table 1.** Frequency of the various mitochondrial clinical syndromes among enrolled participants as of December 31, 2019

Clinical syndromes	Totals	Percent (%)
Alpers syndrome	23	1.5
Cardiomyopathy	12	0.8
CPEO	56	3.7
CPEO “plus”	60	3.9
Diabetes and deafness	24	1.6
Kearns-Sayre syndrome	39	2.6
LHON	42	2.8
Leigh syndrome	189	12.4
Maternal-inherited deafness	9	0.6
MELAS	109	7.1
MNGIE	18	1.2
Multi-systemic syndrome	290	19.0
MERRF	24	1.6
Myopathy	81	5.3
NARP	17	1.1
Pearson syndrome	21	1.4
Reversible infantile myopathy with cytochrome c oxidase deficiency	3	0.2
SANDO	24	1.6
Barth syndrome	3	0.2
Encephalomyopathy	95	6.2
Hepatocerebral syndrome	8	0.5
Leukoencephalopathy	9	0.6
Encephalopathy	77	5.0
Other clinical syndrome/symptoms	296	19.4

cPEO: chronic progressive external ophthalmoplegia; SANDO: sensory ataxic neuropathy with dysarthria and ophthalmoparesis; NARP: neuropathy, ataxia, and retinitis pigmentosa; MERRF: myoclonus epilepsy with ragged red fibers; MNGIE: mitochon-drial neurogastrointestinal encephalopathy; MELAS: mitochondrial encephalomyopathy, lactic acidosis, and stroke-like episodes; LHON: Leber hereditary optic neuropathy

**Table 2.** NAMDC-funded pilot projects

RDCRN protocol number	Pilot project title	Project principal investigator/site
NAMDC7407	Prototype development of an exome variant analysis pipeline and public interface for the community-wide Mitochondrial Disease Sequence Data Resource (MSeqDR)^[2–4]^	Marni Falk, MD, Children’s Hospital of Philadelphia
NAMDC7408	Natural history of pearson syndrome	Sumit Parikh, MD, Cleveland Clinic
NAMDC7416	Citrulline supplementation for treatment of nitric oxide deficiency in MELAS: a Phase 1 dose-finding and safety study	Fernando Scaglia, MD, Baylor College of Medicine
NAMDC7415	The clinical utility and a clinician’s guide to new mitochondrial functional tests^[1]^	JohanL. K. Van Hove, MD, PhD
NAMDC7417	Activators of AMPK for Treatment of Mitochondrial Disorders	Tina M. Cowan, PhD, Stanford University
NAMDC7418	Genomic testing for molecularly undefined NAMDC Registry cases	Amel Karaa, MD, Massachusetts General Hospital
NAMDC7420	The use of amino acids to enhance the activity of enzymes involved in mitochondrial translation defects: a possible therapeutic approach	Marisa Friederich, PhD, University of Colorado
NAMDC7421	Development of Minimally Invasive Nanosensor Technology to Quantify Mitochondrial Function in Human Muscle	Zarazuela Zolkipli Cunningham, MBChB MRCP, Children’s Hospital of Philadelphia

NAMDC: North American Mitochondrial Disease Consortium; RDCRN: Rare Diseases Clinical Research Network; AMPK: adenosine monophosphate–activated protein kinase; MELAS: mitochondrial encephalomyopathy, lactic acidosis, and stroke-like episodes
