# Supplementary material for: The North American mitochondrial disease registry
Source: J Transl Genet Genom. Author manuscript; Available in PMC 2020 Jun 29. (PMC7323997; doi:10.20517/jtgg.2020.12)
Supplement: supplementary materials [file NIHMS1589101-supplement-supplementary_materials.pdf]

Supplemental Tables/Figures

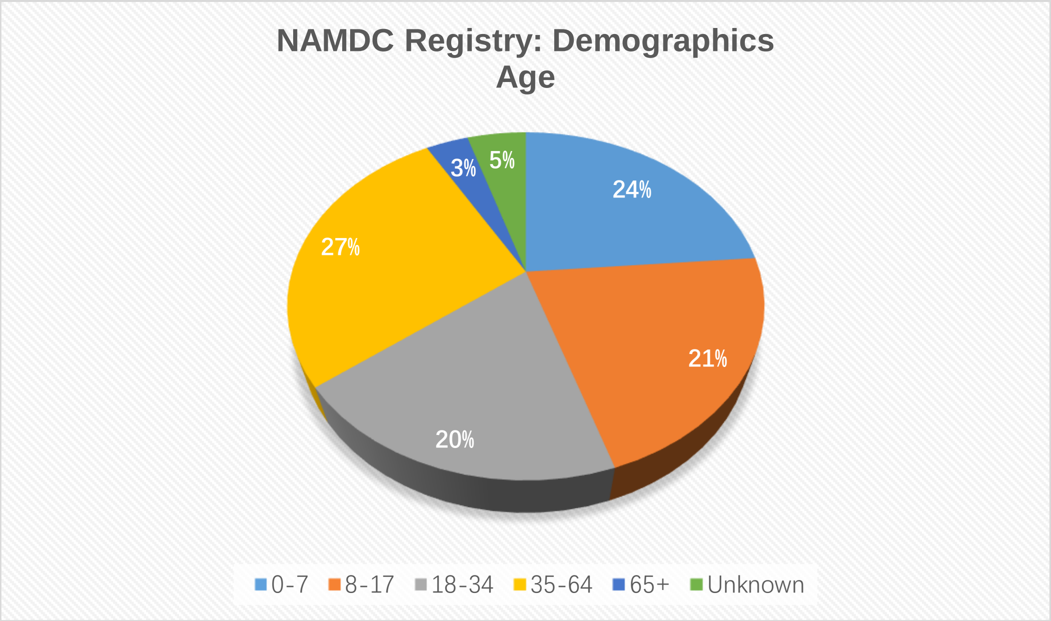

Figure S1. NAMDC Registry Participants by Age group.

Table S1. NAMDC Registry Participants by Ethnicity

	Females	Males	Unknown	Total
**Not Hispanic/Latino**	795 (48.3%)	583 (35.4%)	0 (0.0%)	1378
**Hispanic/Latino**	109 (6.6%)	81 (4.9%)	0 (0.0%)	190
**Not yet reported**	0 (0.0%)	0 (0.0%)	79 (4.8%)	79
**Total**	904 (54.9%)	664 (40.3%)	79 (4.8%)	1647 (100.0%)

Table S1. NAMDC Registry Participants by Race

	Females	Males	Unknown	Total
**American Indian/ Alaska Native**	4 (0.2%)	6 (0.4%)	0 (0.0%)	10(0.6%)
**Asian**	22 (1.3%)	23 (1.4%)	0 (0.0%)	45(2.7%)
**Native Hawaiian/Pacific Island**	4(0.2%)	1(0.1%)	0 (0.0%)	5(0.3%)
**Black/African American**	24 (1.5%)	16(1.0%)	0 (0.0%)	40(2.4%)
**White**	787 (47.8%)	554(33.6%)	0 (0.0%)	1341(81.4%)
**More than one**	16 (1.0 %)	30 (1.8%)	0 (0.0%)	46(2.8%)
**Other**	47 (2.9%)	34(2.1%)	0 (0.0%)	81(4.9%)
**Not yet reported**	0 (0.0%)	0 (0.0%)	79 (4.8%)	79(4.8 %)
**Total**	904 (54.9%)	664 (40.3%)	79 (4.8%)	1647 (100.0%)
